# Characterization of the IFN-γ T-cell responses to immediate early antigens in humans with genital herpes

**DOI:** 10.1186/1743-422X-3-54

**Published:** 2006-07-05

**Authors:** Ralph P Braun, Lendon G Payne, Lichun Dong

**Affiliations:** 1 Wyeth Vaccine Research, 401 North Middletown Rd. Pearl River NY, 109654, USA; 2 Burnett College of Biomedical Sciences, University of Central Florida, Orlando, FL, USA; 3 University of Washington, Dept. of Medicine, 300 9th Ave, Seattle, WA 98104, USA; 4 PowderJect Vaccines Incorporated, 8551 Research Way Boulevard, Middleton, Wisconsin 53562, USA

## Abstract

**Background:**

The IFN-γ ELISPOT assay has been used to examine the T-cell repertoire for many disease states in humans but, as yet, not genital herpes. Using overlapping synthetic peptide libraries, an IFN-γ ELISPOT assay was established that could measure CD4 and CD8 T-cell responses to HSV-2 antigens in patients with genital herpes.

**Results:**

In unexpanded T-cells isolated from peripheral blood, CD4 responses were readily measured against four immediate early antigens (ICP0, ICP4, ICP22 and ICP27), VP22 and gD. The CD4 responses were characterized by a low number of positive cells which produced large ELISPOTs. CD4 responses had a broad specificity and within individual patients several of the test antigens were recognized. In contrast, CD8 responses were found only in approximately 50% of patients and were typically specific to a single antigen. When disease status and immune responses were compared, an enhanced CD4 response to ICP4 in patients with a low recurrence rate was found. The ICP4 response was striking in three HSV-1 single positive genital herpes patients.

**Conclusion:**

The survey of T-cell responses is an important step to understand the host cellular immune response in individuals with genital herpes. The assay described here has the capability of measuring CD4 and CD8 T-cell responses that may be used to correlate disease status with specific immune responses. In an evaluation of 18 subjects a trend of positive responses to an immediate early protein, ICP4, was found in individuals that had a low rate of disease recurrence.

## Background

Genital herpes is a highly prevalent sexually transmitted disease found world-wide and is considered to be a major health burden [1,2]. The causative agent is usually Herpes simplex virus type 2 (HSV-2) although genital herpes caused by the closely related HSV-1 is becoming more prevalent [3,4]. Transmission of virus is primarily through sexual contact and after the initial acute disease a latent infection is established in the dorsal root ganglia of the sensory neurons. From the latent state, the virus can re-activate causing recurrent disease and virus shedding [5,6]. Both antibody and cellular responses are important to control HSV [7,8]. Although antibody responses are able to neutralize virus and reduce disease in animals and humans, they do not provide sterilizing immunity. As well, once a latent infection has been established the presence of high levels of antibody does little to protect against virus reactivation or recurrent disease. Cellular immune responses to HSV are of interest because it is believed that a strong cellular response in addition to an antibody response is important for optimal prevention of HSV disease. Furthermore, it may be the cellular response that is most crucial to control recurrent disease[9-11].

For chronic disease states such as genital herpes, it is important to evaluate the specificity of the T-cell response. Identification of antigens recognized by the immune system is an important description of the character of the immune response. Being able to correlate a specific pattern of immune response to the disease state may allow identification of the type and specificity of the T-cell responses that hold particular importance for control of the disease. A full understanding of the cellular immune responses in humans infected with HSV is not available. Only recently have reports on the detailed specificity of the T-cell response to HSV-2 begun to appear. These have used cloned T-cell lines to define specificities of several CD4 and CD8 T-cells[7,12,13]. Previous work was able to identify cytotoxic responses to several HSV antigens[14,15]; however, in humans there has been no correlate of the specificity of immune responses with the control of the disease.

Of the many methods to measure cellular immune responses, the IFN-γ ELISPOT assay in particular has been found to be an extremely versatile assay[16,17]. The IFN-γ ELISPOT assay is able to measure immune responses with high precision and sensitivity, and is ideal for screening purposes. The assay identifies T-cells that recognize antigens of interest by stimulating cultured T-cells with antigen and measuring a response by the secretion of the cytokine IFN-γ. Since IFN-γ has been identified as an important component of the protection against HSV[18,19], measurement of the IFN-γ response has relevance to control of HSV disease. Recent developments in peptide synthesis have allowed antigens of interest to be synthesized as a library of small peptides whose sequences span the entire protein. Construction of peptides with overlapping sequences can yield libraries that have a high probability of containing any epitope of interest. Because the libraries are not generated to produce epitopes for specific MHC alleles, these libraries can be used to test T-cells from any genetic background or species. These libraries have facilitated a rapid identification of immune responses for many antigens.

Considering that HSV codes for at least 75 potential antigens, some selection of targets for testing must be made. The immediate early antigens are of interest because they are considered to be a group of proteins in which CD8 responses may be generated and the CD8 response in particular may be important for control of HSV[9,20,21]. The immediate early antigens are also of interest as potential vaccine antigens because they appear at the start of a replicative cycle and responses to them could act early in infection. VP22 is a structural antigen that is expressed later in the infection, and unlike the immediate early antigens is present on the virus particle. Both CD4 and CD8 responses have been identified against VP22 in humans[12,22]. Another late antigen of interest is gD which has been studied extensively as a HSV antigen because of the protective antibody response generated by gD in animal models [23,24]. Thus, a comparison of the responses to the immediate early antigens and the late antigens (VP22 and gD) should further delineate the differential cell-mediated immune responses in humans.

## Results

### Detection of HSV-2 specific responses in CD4 and CD8 cell populations

Initial experiments were able to identify HSV-2 specific responses in unfractionated PBMC samples using an IFN-γ ELISPOT assay (data not shown). To characterize the CD4 and CD8 T-cell populations, PBMC samples were depleted of either CD4 or CD8 cells using magnetic beads and the remnant cells designated as CD4 cells (CD8 depleted PBMC sample) and CD8 cells (CD4 depleted PBMC sample) were assayed by an IFN-γ ELISPOT assay (Table 2). The peptide dose needed to stimulate the T-cells was different for the two populations. For CD4 cells, a dose of 0.33 ug/ml of each individual peptide within the pool (defined as 1X) was effective (Table 2) and could be lowered 10 fold without loss of activity (data not shown). In contrast, CD8 cells required a minimum of 3.3 ug/ml dose of each individual peptide (defined as 10X) to stimulate IFN-γ secretion.

**Table 2:** HSV-2 specific responses in the CD4 and CD8 cell populations. Number of ELISPOTs per 500,000 original PBMCs measured in samples depleted of either CD8 cells (designated CD4 cells) or depleted of CD4 cells (designated as CD8 cells) stimulated with various peptide pools in the IFN-γ ELISPOT assay. The peptide pools are described in Table 1 and the 1X dose of peptide is approximately 0.33 ug/ml of each individual peptide within the pool and the 10X dose is approximately 3.3 ug/ml of each peptide within the different pools. Control peptide is a single negative peptide. Sample was from subject 3.

	**CD4 cells**		**CD8 cells**	
**Peptide pools**	**1X dose**	**10X dose**	**1X dose**	**10X dose**

ICP27 all	15	2	0	0
ICP22 all	0	3	0	1
ICP0–1	0	2	0	0
ICP0–2	34	6	0	0
ICP0–3	6	0	0	1
ICP4–1/2	3	0	0	0
ICP4–3/4	45	26	0	43
ICP4–5/6	35	14	0	128
VP22	89	5	0	6
gD	47	25	0	0
Control peptide	0	0	0	0

The CD4 cell population showed positive responses to several peptide pools at the 1X and 10X peptide doses, although the responses were reduced when the 10X dose of peptide was used (Table 2). When the PBMC sample was depleted of CD4 cells and assayed with the 1X peptide dose (Table 2: CD8 cells, 1X dose) no ELISPOTs were detected. This verifies that the cells secreting IFN-γ at the 1X dose level are CD4 cells and not another cell population. In this particular sample a weak CD4 response (defined as below 25 ELISPOTs per ½ million cells) was found against ICP27, and a strong response to pool 2 of ICP0, pools 3/4 and 5/6 of ICP4, VP22 and gD. The responses to pool 3 of ICP0 and pool 1/2 of ICP4 were not considered positives as these responses were not seen upon a repeat assay of this sample. A positive CD8 response (Table 2) was found for ICP4 pools 3/4 and 5/6 when the 10X dose of peptides was used. Intracellular cytokine staining assayed by Flow cytometry using the same peptide pools, and run in parallel with the ELISPOT assay, confirmed that the responses measured were from the CD8 population (data not shown).

**Figure 1:**
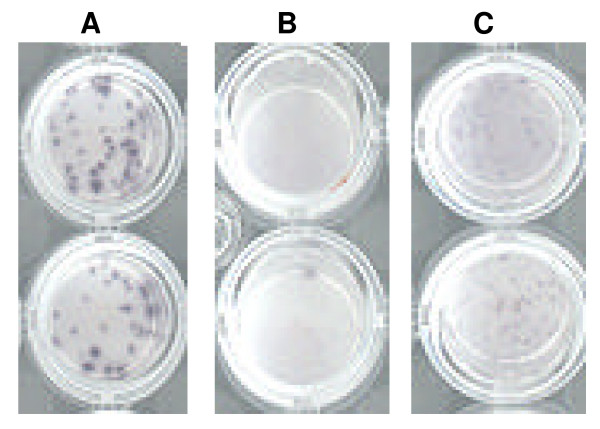
**Comparison of ELISPOTs generated by CD4 and CD8 cells**. CD4 ELISPOTs were detected in a CD8 depleted PBMC sample that had been stimulated with a 1X dose of gD peptides (A). CD8 ELISPOTs were assayed in a CD4 depleted PBMC sample stimulated with either a 1X (B) or 10X (C) dose of an ICP4 peptide pool.

One feature of CD4 ELISPOTs is that they are typically very large (Figure 1A) and can easily be seen without a microscope. The CD8 cell population in the IFN-γ ELISPOT assay was not responsive to the 1X dose of peptide (Figure 1B) and required the higher 10X dose for stimulation (Figure 1C). CD8 ELISPOTs were typically smaller than the CD4 ELISPOTs.

### Optimization of the IFN-γ ELISPOT assay

The IFN-γ ELISPOT assay was refined by using the CD8 population that had been bound to the magnetic beads and physically separated from remnant CD4 cells since both of these cell populations are still functional in an IFN-γ ELISPOT assay. This reduces by one half the amount of blood needed for the assay which is of great value for cellular assays where the amount of sample is usually limiting. The IFN-γ ELISPOT assay was, therefore, split into two separate assays. The CD4 IFN-γ ELISPOT assay used a PBMC sample depleted of CD8 cells as the source of T-cells whereas the CD8 IFN-γ ELISPOT assay tested CD8 cells that had been positively selected onto magnetic beads. Several experiments were done to confirm the validity of the procedure and to ensure that positive selection of CD8 cell populations on magnetic beads yields a cell fraction with comparable activity to unfractionated PBMCs.

**Figure 2:**
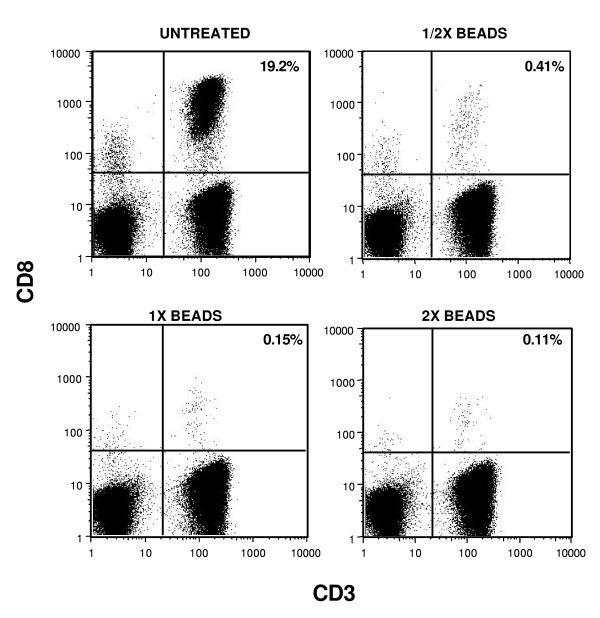
**Flow cytometry of CD8 T-cell populations in PBMC samples with and without depletion of CD8 cells by magnetic beads**. PBMC samples were depleted of CD8 cells using 1/2X, 1X and 2X the manufacturers recommended amount of beads (approximately 10 beads per CD8 cell) prior to Flow cytometry. The percent of total cells that are CD3/CD8 positive is indicated.

Flow cytometric tracking of T-cell populations in PBMC samples before and after magnetic bead depletion (Figure 2) showed that the depletion was very effective. Before depletion of CD8 cells with magnetic beads, the PBMC sample had a CD8/CD3 double positive population of approximately 19% of total cells. When the CD8 T-cells were magnetic bead depleted by 1X (manufacturers recommendation), 2X or 1/2X bead loads, the proportion of CD8 cells remaining were 0.15%, 0.11% and 0.41%, respectively, thus resulting in a 99% depletion. In another PBMC sample, 97% of the CD8 cells were removed when using the 3 different amounts of beads. Thus, magnetic bead depletion of PBMC samples is expected to remove greater than 95% of the CD8 cells and significant cross contamination of T-cells in the two different IFN-γ ELISPOT assays is very low.

**Figure 3:**
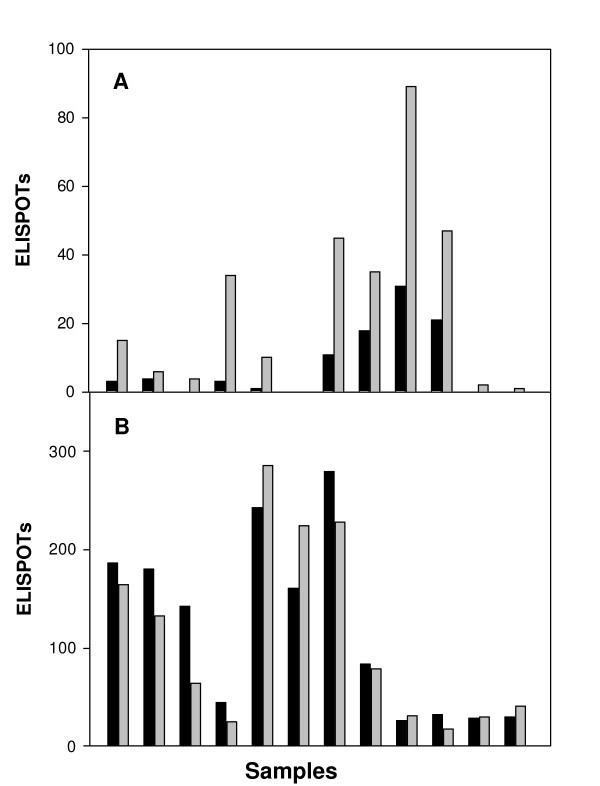
**Comparison of responses by bound and free CD4 and CD8 T-cells**. PBMC samples were treated with magnetic beads specific for CD4 or CD8 cells. Bound CD4 and CD8 cells (black bars) and free cells CD4 and CD8 cells (grey bars) were assayed by the IFN-γ ELISPOT assay. CD4 samples were tested against a library of 12 different peptide pools (Panel A). CD8 samples were tested under various experimental conditions against select positive peptide pools (panel B). Number of ELISPOTS/well is plotted on the Y-axis. The amount of cells placed in each well are those recovered from an original 0.5 million PBMCs.

To evaluate the activity of T-cells that were bound to magnetic beads, PBMC samples were depleted of either CD4 or CD8 cells and then both the beads and the remnant cells were tested in the IFN-γ ELISPOT assay (Figure 3). For the CD4 responses, one PBMC sample that had been divided into bead-bound CD4 cells and free CD4 cells was tested against a panel of 12 different peptide pools. The ELISPOTs measured in the free CD4 cells were always higher than the assay done with bead-bound CD4 cells. Total ELISPOTs from all wells was 92 for the bead-bound CD4 cells and 288 for the free CD4 cells, indicating that CD4 cells positively selected onto magnetic beads do not have the same activity as CD4 cells within a PBMC sample.

For assay of CD8 responses, PBMC samples from patients known to have positive responses were fractionated into bead-bound CD8 cells and free CD8 cells and tested against positive peptide pools (ICP4 or VP22) under a variety of experimental conditions. Twenty-four of these side-by-side comparisons yielded very similar results (Figure 3B). The total ELISPOTs counted from the 24 different replicates was 1892 for the bead-bound CD8 cells and 1717 for free CD8 cells indicating that the bead bound CD8 cells and the free CD8 cells generate the same response under these assay conditions. The ability of CD8 cells to respond to peptide when bound to beads suggests that antigen presenting cells are not required or are not limiting under the conditions of the assay. In support of this, experiments in which antigen presenting cell populations were either added or removed had no effect on the CD8 IFN-γ ELISPOT assay (data not shown).

### Survey of T-cell responses from infected subjects

PBMCs from a total of 18 individuals were tested using the CD4 and CD8 IFN-γ ELISPOT assays in which the CD4 response was measured in CD8 depleted samples and the CD8 response was measured by CD8 cells bound to magnetic beads. A panel of several HSV-2 antigens were tested. Information was gathered on their use of antivirals, recurrence rates (based on subjects recollection but not confirmed medically), sex and age. A commercial serological test was used to determine serostatus of the patients (Table 3). No HLA typing was performed and because of this no attempt was made to define epitopes in positive peptides.

**Table 3:** Subject characteristics. Individual characteristics of subjects used in this study. Subjects are numbered by the order of first blood collection. Some subjects had 2 or 3 blood draws. Recurrence rates are based on patients recollection and were divided into groups of low recurrence rates (L) of 2 or fewer per year and high recurrence rates (H) for subjects that stated having more than 2 recurrences a year. Positive designation for antivirals are those patients that were using antivirals at the time of blood draw. To determine serostatus the HerpeSelect 1 & 2 immunoblot kit from Focus technologies was used. Patient 4 is a double negative. Subjects 6 and 15 were using acyclovir and subject 11 was using daily doses of Valtrex.

Subject	Sex	Age	Antiviral	Recurrence rate	Serostatus
1	M	37	-	H	HSV-2/HSV-1
2	F	38	-	L	HSV-2/HSV-1
3	F	47	-	H	HSV-2/HSV-1
4	M	29	-	na	-
5	M	34	-	H	HSV-2/HSV-1
6	F	49	+	H	HSV-2
7	F	22	-	L	HSV-1
8	F	42	-	H	HSV-2
9	F	24	-	L	HSV-2
10	F	19	-	L	HSV-1
11	F	29	+	L	HSV-2/HSV-1
12	M	28	-	H	HSV-2
13	F	40	-	L	HSV-2
14	F	38	-	H	HSV-2
15	M	59	+	H	HSV-2/HSV-1
16	F	45	-	L	HSV-2
17	M	27	-	L	HSV-1
18	M	53	-	L	HSV-2

The CD4 IFN-γ ELISPOT assay results were tabulated using a relative strength of response with 25 or more positive spots per one half million cells being a strong response and a weak response were those above the background but below and up to 25 spots per one half million cells (Table 4a). For CD8 responses, only those responses that were positive are indicated (Table 4b). Only positive responses verified by a second assay were considered positives. Subject 4 is a double negative patient based on the serotyping assay and did not have any positive responses in the IFN-γ ELISPOT assay. It is readily apparent from Table 4, that the CD8 responses are narrow and focused on small numbers of antigens, whereas the CD4 responses recognize a broad range of antigens.

**Table 4a:** CD4 IFN-γ ELISPOT assay results. CD4 responses measured by the CD4 IFN-γ ELISPOT assay were graded based on their strength into either negative (blank), weak (W) of less than 25 ELISPOTs/0.5 million PBMCs or strong (S) of 25 or more ELISPOTs/0.5 million PBMCs.

**CD4 RESPONSES**													
Subject	ICP27	ICP22	ICP0–1	ICP0–2	ICP0–3	ICP4–1	ICP4–2	ICP4–3	ICP4–4	ICP4–5	ICP4–6	VP22	gD

1	**S**	**S**						W	W	W	W	**S**	W
2			W					**S**		**S**			**S**
3	W			**S**					**S**	**S**	**S**	**S**	**S**
4													
5	W	W			**S**				W				**S**
6	W	W		**S**		W	W		W			**S**	**S**
7								**S**		W			
8		W	**S**					W		W		W	W
9	W			**S**		W		**S**	W	**S**		W	W
10					W					**S**			
11	W	W					W	W				**S**	**S**
12		**S**	**S**		W		W	W	W	**S**		**S**	**S**
13					**S**							**S**	**S**
14													**S**
15		W	**S**					W		W	W	**S**	**S**
16			W					**S**				W	**S**
17								**S**		**S**			
18		W		**S**			W	W	**S**		**S**	**S**	W

**Table 4b:** CD8 IFN-γ ELISPOT assay results. CD8 responses measured by the CD8 IFN-γ ELISPOT assay were designated as either negative (blank), or positive (P).

**CD8 RESPONSES**													
Subject	ICP27	ICP22	ICP0–1	ICP0–2	ICP0–3	ICP4–1	ICP4–2	ICP4–3	ICP4–4	ICP4–5	ICP4–6	VP22	gD

1													
2												**P**	
3									**P**	**P**	**P**		
4													
5				**P**								**P**	
6													
7													
8				**P**									
9					**P**					**P**			
10													
11													
12													
13													
14												**P**	
15												**P**	
16													
17													
18												**P**	

### CD8 responses

#### Definition of CD8 responses

Eight of the 17 infected subjects had positive CD8 responses with PBMCs from only two individuals recognizing more than a single antigen. Responses were found to VP22, ICP0 and ICP4. No CD8 responses were identified for ICP27, ICP22 or gD in the population tested. The specific peptides responsible for the responses were determined by reassaying PBMCs from samples with sufficient material with peptide pools containing fewer peptide species. In two individuals with positive responses to VP22, an epitope was localized to the same two overlapping peptides, RGAGPMRA**RPRGEVRFL**HY and **RPRGEVRFL**HYDEAGYALY. These two peptides both contain the RPRGEVRFL sequence that is one of the few known CD8 epitopes for HSV-2[12]. A response against ICP0 was also narrowed to a single peptide but did not contain any sequences previously described as a CD8 epitope.

#### Dose and length dependence of CD8 peptides

**Figure 4:**
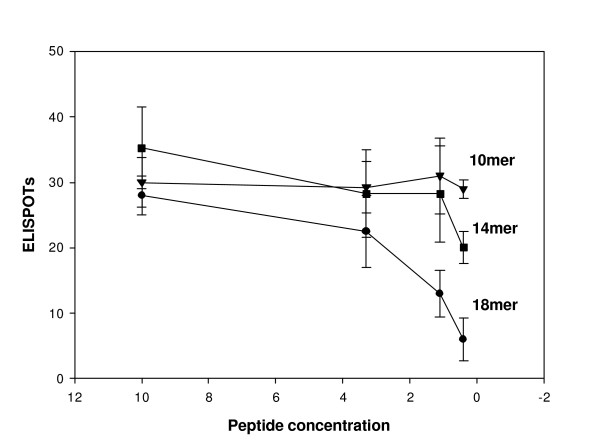
**Effect of peptide length on responses CD8 responses**. CD8 cells isolated on magnetic beads from a subject with positive responses to VP22 were tested in the IFN-γ ELISPOT assay against peptides of various lengths each containing the putative RPRGEVRFL epitope. Results are averages of two experiments run in duplicate using CD8 cells harvested from 0.3 million PBMCs per well. Frozen PBMCs were used for this experiment.

The peptide pools were composed of peptides of 18 or 19 amino acids in length, which is longer than needed for CD8 epitopes [25]. The increased length may be responsible for the high dose requirements of the CD8 assay. Once a positive epitope was identified, a series of peptides of various lengths were synthesized to examine the effect of peptide length on CD8 responses. Peptides of 10, 14 or 18 amino acids in length were synthesized to contain the epitope defined for VP22. Using concentrations of 10, 3, 1 and 0.3 ug/ml of peptides, the response of subject 14 to the different peptide doses was measured (Figure 4). Similar responses were found for the three peptides at high doses, however, as the dose was reduced below 3 ug/ml the 18mer peptide began to lose activity. The 10mer peptide maintained activity at all doses, whereas the 14mer showed an intermediate activity suggesting that the dose dependency of the CD8 IFN-γ ELISPOT assay is related to the length of the peptides. Although longer peptides may be less active at low doses, the concentration of peptide used for the CD8 IFN-γ ELISPOT assay is sufficient to identify CD8 responses using long peptides.

#### EBV CD8 responses

**Figure 5:**
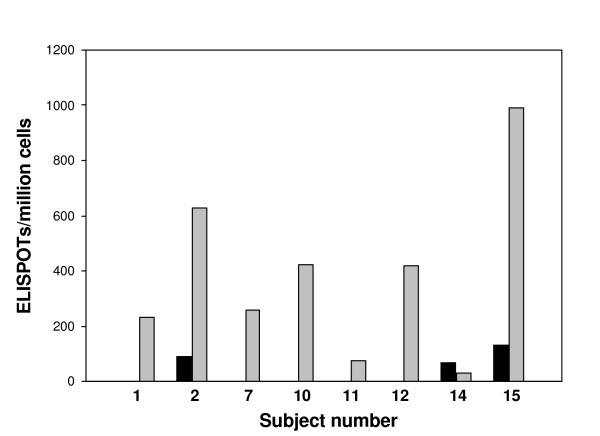
**EBV and HSV-2 specific CD8 responses**. Frozen PBMC samples were treated with magnetic beads to positively select for CD8 cells and the same preparation was tested against the known positive HSV-2 peptide pools for that subject (black bars) and the EBV CD8 epitope peptide pool (grey bars) using the IFN-γ ELISPOT assay.

Since very few positive CD8 responses were identified in the samples tested, an additional control was used to assess the capability of the samples, and the assay, to measure CD8 responses. EBV infection is very common in humans and the CD8 response to several antigens have been defined [26]. A pool of peptides representing CD8 epitopes for EBV was used to compare EBV specific CD8 responses with HSV-2 specific CD8 responses within the same PBMC samples (Figure 5). Using 8 available frozen samples, responses against the HSV-2 antigens and the EBV peptides were measured with the CD8 IFN-γ ELISPOT assay. Strong positive responses to the EBV pool were detected in 6/8 samples whereas only 3 of these samples were positive for HSV-2 responses. Notably, samples that did not have positive HSV-2 responses could have strong positive CD8 responses to EBV. These results confirm the capability of the CD8 IFN-γ ELISPOT assay to measure CD8 responses and that samples negative for HSV-2 specific CD8 responses are still active. The average strength of the ELISPOT positive responses was greater for the EBV antigens (115 spots) than to HSV-2 antigens (30 spots).

### CD4 responses

#### Specificity

CD4 responses were found to have a broad specificity with positive responses to multiple antigens in all patients (see Table 4A). Responses to VP22, gD, ICP4 and ICP0 were consistently strong and found in most patients. The CD4 responses to ICP27 and ICP22 were generally weaker and less common than the other antigens.

#### Stability of CD4 responses

**Figure 6:**
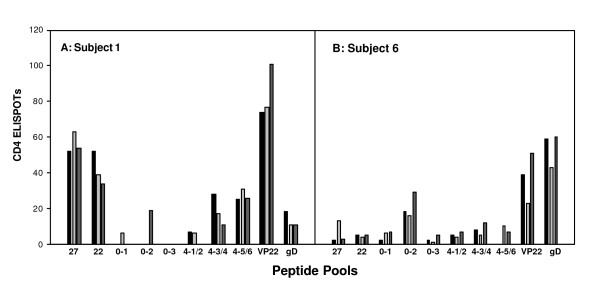
**Longitudinal CD4 responses**. Blood was taken approximately 1 month apart from 2 subjects (3 blood draws in total) and a PBMC sample was frozen from each blood draw. PBMCs from all blood draws were then thawed and tested at the same time in the CD4 IFN-γ ELISPOT assay. The number of ELISPOTS/well is plotted on the Y-axis. The amount of cells placed in each well are those recovered from an original 0.5 million PBMCs.

The stability of the strength and specificity of the CD4 responses to the different antigens was examined in sequential blood samples. Three blood samples were obtained from two individuals at approximately 1-month intervals and the CD4 responses were tested (Figure 6). These two individuals did not have positive CD8 responses. When comparing responses measured in fresh PBMC samples for the 3 bleeds, the pattern of positive and negative pools remained the same (data not shown). When frozen samples were used for simultaneous quantitative comparison on the same ELISPOT plate, the values were similar. Neither subject reported a recurrence during the time between the blood draws. Thus, the strength and specificity of the CD4 responses in individuals was found to be stable over time.

### Comparison of T-cell responses and subject characteristics

One goal of studying the immune responses from infected individuals is to correlate disease severity to immune responses. Although the mixed population of 17 infected subjects studied here represents a small sample size, some trends were apparent. When immune response measures and disease status were compared in the test population a trend in the CD4 response to ICP4 and disease recurrence was found (Table 5). When subjects were divided into those with low recurrence rates of 2 or fewer per year, and those with rates above 2 per year, the low recurrence group showed a higher CD4 response to ICP4. In contrast, all other antigens had a low CD4 response when subjects had few recurrences. To quantitate this effect the number of all ELISPOTs (E#) measured for each peptide pool was totalled and listed in Table 5. The ELISPOTs for each protein were totalled and the ratio of the ELISPOTs from the low recurrence subjects to the high recurrence individuals was calculated (Table 5). Only the response to ICP4 had a ratio that was greater than 1.0. The subjects with stronger CD4 responses to ICP4 tend to show weak responses to VP22 and gD; two antigens that are generally strong. In individuals with low recurrence rates, the total number of CD4 ELISPOTs for ICP4 was approximately 3 times more than to VP22, whereas the high recurrence group shows roughly the same number of ELISPOTs for these two antigens. Although not as apparent as with ICP4, the CD4 responses to ICP0 also seem to follow the same trend of greater strength than VP22 responses in subjects with low recurrences.

**Table 5:** T-cell responses for subjects grouped by rate of recurrences. Data is the same as Table 2 but subjects have been organized into groups based on the rate of recurrent disease. For CD4 responses, the total of the number of ELISPOTs (E#) for each peptide pool is included. Total number of CD4 ELISPOTs for each protein from each group were used to generate a ratio of the ELISPOTs measured in low recurrence subjects compared to high recurrence subjects. Values were adjusted to compensate for the different number of subjects per group. A second calculation was done in which three subjects (7, 10, 17) that were single positive for HSV-1 were removed.

**HIGH RECURRENCES PER YEAR (>2)**																	
**CD4 Responses**														**CD8 Responses**			

Subject	ICP27	ICP22	ICP0–1	ICP0–2	ICP0–3	ICP4–1	ICP4–2	ICP4–3	ICP4–4	ICP4–5	ICP4–6	VP22	gD		ICP0	ICP4	VP22

1	**S**	**S**						W	W	W	W	**S**	W				
3	W			**S**					**S**	**S**	**S**	**S**	**S**			**P**	
5	W	W			**S**				W				**S**		**P**		**P**
6	W	W		**S**		W	W		W			**S**	**S**				
8		W	**S**					W		W		W	W		**P**		
12		**S**	**S**		W		W	W	W	**S**		**S**	**S**				
14													**S**				**P**
15		W	**S**					W		W	W	**S**	**S**				**P**
E#	80	102	121	80	49	9	13	61	112	118	56	374	475				

**LOW RECURRENCES PER YEAR (≤2)**																	

**CD4 Responses**														**CD8 Responses**			

Subject	ICP27	ICP22	ICP0–1	ICP0–2	ICP0–3	ICP4–1	ICP4–2	ICP4–3	ICP4–4	ICP4–5	ICP4–6	VP22	gD		ICP0	ICP4	VP22

2			W					**S**		**S**		W	**S**				**P**
7								**S**		W							
9	W			**S**		W		**S**		**S**		W	W		**P**	**P**	
10					W					**S**							
11	W	W					W	W				**S**	**S**				
13					**S**							**S**	**S**				
16			W					**S**				W	**S**				
17								**S**		**S**							
18		W		**S**			W	W	**S**		**S**	**S**	W			**P**	
E#	21	30	45	78	75	5	14	354	46	190	31	196	187				

	ICP27	ICP22	ICP0	ICP4	VP22	gD											
	0.23	0.26	0.70	1.54	0.47	0.35	Low/high recurrence ratio including HSV-1 single positives										
	0.35	0.39	1.02	1.49	0.69	0.52	Low/high recurrence ratio without HSV-1 single positives										

It is noteworthy that Subject 11 (Table 5) was the only subject that was taking a daily regimen of Valtrex. This individual reported two recurrences per year and thus, was included in the low recurrence group. However, the pattern of the CD4 response (stronger responses to VP22 and gD, weaker response to ICP4) appears to be more like that of the high recurrence individuals. Thus, for this individual the use of daily antiviral may be responsible for the reduction in the number of recurrences rather than the immune response pattern typical of subjects that have a high number of recurrences.

The correlation of immune responses and recurrence rates was reanalyzed by grouping subjects with respect to their HSV serostatus, since this can correlate with disease severity and recurrence rates [27]. The subjects tested showed a slight trend of more recurrences in HSV-2 single positive subjects, fewer recurrences among HSV-1/HSV-2 double positive individuals and the fewest recurrences in HSV-1 single positives (data not shown). Comparison of the strength of CD4 responses (Table 6), however, did not show a clear trend relative to serostatus. Thus, stronger ICP4 and weaker VP22 and gD CD4 responses correlate better with the rate of recurrences than with serostatus.

**Table 6:** T-cell responses for subjects grouped by HSV-1/HSV-2 serostatus. Data is the same as Table 2 but subjects have been organized into groups based on their HSV-1 and HSV-2 serostatus. For CD4 responses, the total number of ELISPOTs (E#) for each peptide pool of that group is included. Total number of CD4 ELISPOTs for each protein from each group were used to generate a ratio of the ELISPOTs measured in HSV-1/HSV-2 double positives and HSV-2 single positives. Values were adjusted to compensate for the different number of subjects per group

**HSV-2 SINGLE POSITIVIES**																	
**CD4 Responses**														**CD8 Responses**			

Patient	ICP27	ICP22	ICP0–1	ICP0–2	ICP0–3	ICP4–1	ICP4–2	ICP4–3	ICP4–4	ICP4–5	ICP4–6	VP22	gD		ICP0	ICP4	VP22

6	W	W		**S**		W	W		W			**S**	**S**				
8		W	**S**					W		W		W	W		**P**		
9	W			**S**		W		**S**		**S**		W	W		**P**	**P**	
11	W	W					W	W				**S**	**S**				
12		**S**	**S**		W		W	W		**S**		**S**	**S**				
14													**S**				**P**
15		W	**S**					W		W	W	**S**	**S**				**P**
16			W					**S**				W	**S**				
E#	37	87	133	62	21	14	18	135	62	69	8	309	394				

**HSV-2/HSV-1 DOUBLE POSITIVES**																	

**CD4 Responses**														**CD8 Responses**			

Patient	ICP27	ICP22	ICP0–1	ICP0–2	ICP0–3	ICP4–1	ICP4–2	ICP4–3	ICP4–4	ICP4–5	ICP4–6	VP22	gD		ICP0	ICP4	VP22

1	**S**	**S**						W	W	W	W	**S**	W				
2			W					**S**		**S**		W	**S**				**P**
3	W			**S**					**S**	**S**	**S**	**S**	**S**			**P**	
5	W	W			**S**				W				**S**		**P**		**P**
13					**S**							**S**	**S**				
18		W		**S**			W	W	**S**		**S**	**S**	W			**P**	
E#	64	45	23	96	96	0	9	128	96	109	64	261	254				

**HSV-1 SINGLE POSITIVES**																	

**CD4 Responses**														**CD8 Responses**			

Patient	ICP27	ICP22	ICP0–1	ICP0–2	ICP0–3	ICP4–1	ICP4–2	ICP4–3	ICP4–4	ICP4–5	ICP4–6	VP22	gD		ICP0	ICP4	VP22

7								**S**		W							
10					W					**S**							
17								**S**		**S**							
					7			152		130							
	ICP27	ICP22	ICP0	ICP4	VP22	gD											
	2.4	0.68	1.3	1.5	1.1	0.86	Ratio of double positive/HSV-2 single positive										
																	

The three HSV-1 single positive individuals had an extremely skewed CD4 response (Table 6) towards ICP4 and no response to VP22 or gD. To ensure that these individuals were not solely responsible for the immune bias found in Table 5, these patients were excluded and the ratios recalculated. Even after exclusion of these HSV-1 single positive patients the pattern of stronger ICP4 and weaker VP22 and gD CD4 responses was maintained.

### HSV-1 single positive patients

The three single positive HSV-1 subjects (7, 10, 17) had a very distinct pattern of responses to ICP4 with no or weak responses to all other antigens. Interpreting these results is complex since the ICP4 peptide library was based on the sequence of HSV-2 MS strain which has an approximate 60% homology to HSV-1 for the antigens in this region. Thus, it is interesting that not only is ICP4 uniquely recognized in HSV-1 single positive patients but this response appears to be stronger than in HSV-2 positive patients and is specific for pools 3 and 5. When peptide subsets from ICP4 pools 3 and 5 were tested, the reactive peptides were from an HSV-2 sequence region that was identical to HSV-1, thus, explaining the cross reactivity.

**Figure 7:**
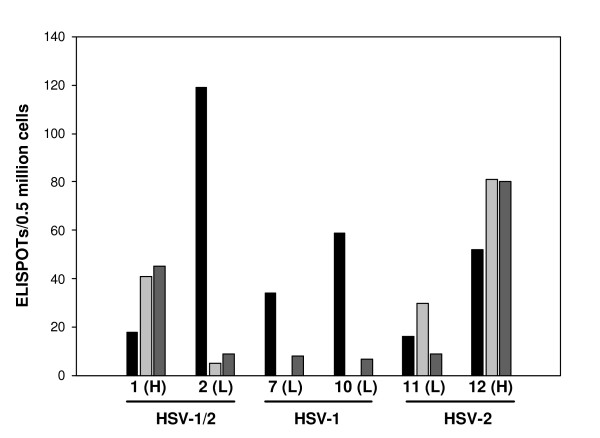
**Comparison of CD4 responses to ICP4, HSV-1 VP22, and HSV-2 VP22**. Frozen samples from several subjects were tested in the CD4 IFN-γ ELISPOT assay using peptide pools for ICP4 (left column), VP22 from HSV-2 (middle column) and VP22 from HSV-1 (right column). Subjects (1,2,7,10,11,12) were chosen based on serostaus (HSV-1/2, HSV-1, HSV-2) and designated as having high (H) or low (L) recurrence rates. Number of ELISPOTS/well is plotted on the Y-axis. The amount of cells placed in each well are those recovered from an original 0.5 million PBMCs.

A library of VP22 HSV-1 peptides was used to determine if the weak responses to HSV-2 VP22 peptides in HSV-1 single positive patients (see Table 6) could be ascribed to homology differences between the virus serotypes. Frozen samples of single positive HSV-2, single positive HSV-1, and double positive subjects were assayed for responses to ICP4, HSV-1 VP22, and HSV-2 VP22 (Figure 7). Even though CD4 responses to HSV-1 VP22 were found in the low recurrence HSV-1 single positives (subjects 7 and 10) and HSV-2 double positives (subject 2), they were much lower than those found to ICP4. Thus, the trend of the immune response of ICP4>VP22 in low recurrence rate individuals was once again demonstrated. The exception is subject 11, who as described earlier was taking Valtrex daily and may not be a true immunologically low recurrence individual. High recurrence individuals HSV double positive subject 1 and HSV-2 positive subject 12 had a response that was VP22>ICP4.

## Discussion

HSV specific antibody immune responses have been well studied in humans based on the hope that a strong neutralizing antibody response may be effective in prophylaxis of HSV infection [28,29]. Although an antibody response by itself may reduce disease, it cannot stop HSV infection. Mounting evidence indicates that the cellular immune response is also necessary for control of HSV. In HIV infected individuals a greater susceptibility to HSV recurrence correlated to a general loss of CD8 responses [10], however, no correlate of disease severity to a specific antigenic response has been found. Information on the specificity of the immune response in individuals with genital herpes is needed to help define protective responses against HSV. Only recently has information become available on the specificity of the cellular HSV response using cloned T-cell lines, but, data from other methods, especially quantitative assays, are still needed. The IFN-γ ELISPOT assay has been used to examine the T-cell repertoire for many disease states in humans but, as yet, not for genital herpes.

To examine the character of the T-cell response in HSV infected individuals, an IFN-γ ELISPOT assay was developed that was capable of measuring responses in CD4 and CD8 T-cell populations. The IFN-γ ELISPOT assay was chosen because it has the sensitivity and specificity needed to obtain a detailed analysis of the T-cell responses. The goal was to establish an assay that was simple, reproducible, and was capable of measuring responses to a large panel of antigens in an individual regardless of genetic background. One advantage of the IFN-γ ELISPOT assay is that it does not require a prior knowledge of epitopes and can be used to evaluate many antigens at one time with very high sensitivity. The survey of T-cell responses described here is an important step to understand the host cellular immune response in individuals with genital herpes. Although this work identified many T-cell responses it is not expected to be comprehensive. HSV specific T-cells that secrete cytokines other than IFN-γ would not have been measured. As well, due to the nature of this technique and the reagents, and variations in the strain of virus infecting subjects, not all responses would be identified.

A key variable for any *in vitro *cellular immune assay is the nature of the antigenic stimulant. Synthetic peptide libraries of 18 or 19 amino acids in length were chosen because they have the potential for stimulating both CD4 and CD8 T-cells and also because of the ease to which positive responses can be narrowed to single peptides. The results indicate that the IFN-γ ELISPOT assays that were established for this study were able to measure IFN-γ secretion from both CD4 and CD8 cells. For CD8 responses, the ability to measure responses in positively selected CD8 cells, and to identify responses to known EBV and HSV-2 CD8 epitopes, indicates the results are measures of authentic CD8 responses. Although it appeared that the length of peptide was not optimal for CD8 responses this was overcome by using high doses of peptide. For the CD4 IFN-γ ELISPOT assay, the loss of ELISPOTs after depletion of CD4 cells by magnetic beads confirmed that the response was from CD4 cells. Considering the efficiency of the magnetic bead separation used to prepare the samples, and the different assay conditions needed for the two T-cell populations, the results are not expected to be measurably affected by cross contamination of the populations.

The ability to measure both CD8 and CD4 responses in unexpanded samples makes the assays described here sensitive measurements of the T-cell responses in HSV infected individuals. The sensitivity would be important for measuring changes that may occur during the course of the disease and during recurrences. Similarly, the effect of therapeutic intervention, such as antiviral treatment or vaccination, can be monitored to differentiate the effect of therapy on pre-existing responses. Furthermore, since IFN-γ is an important cytokine for the control of HSV-2 disease [18,19], the measurement of IFN-γ secretion will be relevant to disease control.

The T-cell responses measured in the 17 HSV infected patients revealed a broad specificity CD4 response and a narrow CD8 response. Our investigation found only CD8 responses to ICP4, ICP0 and VP22. Koelle *et. al*. [12,30] have also found a limited repertoire of CD8 responses in which VP22 and ICP0 were among the recognized antigens. In contrast, a previous study commonly detected CD8 responses to immediate early antigens, especially those specific for ICP27 [14]. However, that study used cells expanded in culture. We have also found that restimulation of cells in culture generates positive responses that were not measurable in unexpanded PBMCs (data not shown) including those to ICP27. This suggests that additional very low-level CD8 responses may be present in PBMCs. However, because expansion of cells may alter the relative levels of the different CD8 cell populations, *in vitro *expansion of cells was not pursued in our studies in order to maintain the ability of the IFN-γ ELISPOT assay to quantitate differences in the T-cell responses.

The CD8 response is considered to be important for clearance of infectious HSV and possibly maintain the virus in the latent state [11,31]. We were unable to find a correlate of CD8 responses with disease severity although only a limited number of responses were identified in this study. The cyclic nature of recurrences in genital herpes suggests that the cause may not be with the presence or absence of a protective response but rather the level of response needed for protection. As virus antigen declines following a recurrent episode the protective cellular response may also decline to below a threshold where the individual becomes susceptible to reactivation. Measuring the quantity of CD8 responses longitudinally in infected individuals may be able to correlate a change in CD8 responses with recurrence of disease. However, the changes in the CD8 response may not be apparent in PBMCs but may be localized in the skin or nervous system. The recent finding that HSV specific T-cells predominantly have the skin homing molecule CLA on their surface supports this possibility [32]. Using the assays and information that have recently become available, highly precise definition of the T-cell responses in individuals with genital herpes will begin to reveal important immunological features of this disease.

The CD4 T-cell responses that were measured were broader than the CD8 responses and all antigens that were tested were recognized by at least one of the subjects. It is noteworthy that the CD4 response could be correlated to the severity of genital herpes. In particular, the CD4 response to ICP4 appeared to be stronger in individuals with a low recurrence rate compared to those who had a high recurrence rate. Although not as apparent as the responses to ICP4, responses to ICP0 also may have a similar trend of increasing within individuals with less severe disease. In contrast CD4 responses to VP22 and gD were stronger in high recurrence rate patients compared to the low recurrence rate patients. Thus, a general trend in individuals who control HSV disease is that the CD4 responses to immediate early antigens (ICP0 and ICP4) are stronger than to antigens produced abundantly later in the virus life cycle such as VP22 and gD. This would not be unexpected as individuals that limit the replication of virus should have less severe disease and would limit the availability of antigens that are produced later in the virus life cycle. It is still open whether or not it is the immune response that determines the disease recurrence rate, or if the recurrence rate affects the immune response. Nevertheless, the CD4 responses to ICP4 and ICP0, we describe here, indicate an availability of these two antigens to the immune system in individuals with low recurrence rates. Thus, ICP4 and ICP0 may be potential targets for therapeutic immunization. The basic premise is that any therapeutic intervention that can alter the bias of the immune response towards immediate early antigens may aid in reducing the disease recurrence and severity.

If further study confirms a bias toward stronger CD4 responses to ICP4 and away from VP22 and gD, then this pattern may be used as an indicator of disease severity. Patients on suppressive antiviral therapy may be of special interest since the severity of their disease is controlled by anti-virals not the immune response. We report here one individual that was treated with anti-viral suppressive therapy had an immune response that remained focused on late antigens indicative of a more severe disease. Because antiviral treatment would limit production of HSV antigen an alteration of the immune response is not expected during therapy. This would indicate that ceasing antiviral treatment would return the individual to the original disease severity.

The finding that stronger CD4 responses to ICP4 were present in individuals with less severe disease suggests that even with few or no recurrences the immune system is being exposed to virus antigen. The strength of the responses would argue that the virus may be continually attempting to reactivate from the latent state but may be controlled sufficiently to suppress or abort a recurrence of active disease. Study of reactivation events in latently infected human and mouse ganglia indicate that during latency the virus is actively attempting to reactivate[33,34].

The mechanism by which an immune response may help control HSV is unknown. Cytotoxic CD4 cells have been described [35,36] and the CD4 cells examined here could act through a lytic mechanism although we have not assessed this. Alternatively, the CD4 cells could act through secretion of IFN-γ [37]. In skin lesions, CD4 cells are believed to help resolve lesions by secretion of IFN-γ that eventually sets the stage for virus clearance by CD8 cells. Thus, even though CD8 cells may play a downstream role in controlling HSV recurrences the CD4 population identified here may play a crucial role by creating a suitable environment with appropriate kinetics.

## Conclusion

An IFN-γ ELISPOT assay has been used to measure CD4 and CD8 T-cell responses in subjects with genital herpes. The assay has the capability of measuring T-cell responses in unexpanded PBMCs and may be useful to correlate disease status with specific immune responses. The evaluation of 18 subjects found a trend of positive CD4 responses to ICP4 in individuals that had a low rate of disease recurrence.

## Methods

### Peptide libraries

Peptides were synthesized as Pepset Libraries (Mimotopes, Fisher Scientific Raleigh NC). Peptides intended to span the complete amino acid sequence of target antigens were 18 or 19 amino acids in length and overlapped adjacent peptides by 11 amino acids. Individual peptides were suspended in DMSO (Sigma-Aldrich, St. Louis, MO) and were used alone or combined to form peptide pools. The sequences used to construct the libraries and different pools are detailed in Table 1. Other peptides such as those from EBV, or the VP22 specific peptides made to various lengths, were also synthesized as part of Pepset Libraries and suspended in DMSO for use. Sequences used for EBV peptides were from Currier *et. al*. [26]

**Table 1:** Description of peptide pools. The number of peptides within a given pool is indicated as well as the region of the protein that the peptide sequences would correspond to. The virus strain that the peptide sequence was based on is indicated.

**Pool**	**Number of peptides**	**Peptides within pool**	**Amino Acid coordinates**	**Virus Strain**	**Peptide size/overlap**
ICP27 (all)	72		1–512	HSV-2 MS	18/11
ICP27–1	36	1–36	1–263		
ICP27–2	36	37–72	253–512		
ICP22 (all)	58		1–414	HSV-2 MS	18/11
ICP22–1	29	1–29	1–213		
ICP22–2	29	30–58	203–414		
ICP0	113			HSV-2 MS	18/11
ICP0–1	37	1–37	1–270		
ICP0–2	37	38–74	260–529		
ICP0–3	39	75–113	519–801		
ICP4	189			HSV-2 MS	18/11
ICP4–1	32	1–32	1–233		
ICP4–2	32	33–64	223–457		
ICP4–3	32	65–96	447–681		
ICP4–4	32	97–128	671–905		
ICP4–5	32	129–160	895–1129		
ICP4–6	29	161–189	1119–1331		
gD	55	1–55	1–300	HSV-2 HG52	19/11
VP22(2)	42	1–42	1–393	HSV-2 HG52	19/11
VP22(1)	42	1–42	1–301	HSV-1 17	19/11

### Sample collection

Blood was collected by Covance laboratories in Madison, WI following established guidelines for blood sampling of human subjects including IRB approval. Subjects were recruited in local STD clinics for individuals with genital herpes. Subjects were not prescreened before blood collection. HSV serostatus of the subjects was determined using the HerpeSelect 1 and 2 Immunoblot IgG kit (Focus Technologies, Cypress, CA) from plasma collected during blood processing.

#### Blood processing

Whole blood was collected in sodium heparin Vaccutainer tubes (Becton Dickinson, Franklin Lakes, NJ) and peripheral blood mononuclear cells (PBMCs) were separated on a density gradient using Accuspin System-Histopaque-1077 tubes (Sigma-Aldrich) with centrifugation at 1,800 × *g *for 30 min. PBMCs were washed twice in RPMI-5 Medium: RPMI-1640 (Bio-Whittaker, Walkersville, MD) supplemented with 5% heat Inactivated fetal bovine serum (FBS) (Harlan, Indianapolis IN), gentamycin (50 μg/ml, GIBCO Invitrogen Corp., Carlsbad, CA) and antibiotic/antimycotic (1%, GIBCO Invitrogen Corp.). Following the washing steps, cells were counted and assayed for ELISPOT activity.

##### ELISPOT assays

PBMCs were suspended at 5 million cells/ml in RPMI-10 media; RPMI-1640 supplemented with 10% heat-inactivated Human AB Serum (Pel-Freeze, Rogers, AR) antibiotics/antimycotic (1%), gentamycin (50 μg/ml), sodium pryuvate (1 mM, GIBCO Invitrogen Corp.) non-essential aa's (1%, GIBCO Invitrogen Corp.) and β-mercaptoethanol (50 μM, GIBCO Invitrogen Corp.).

##### Magnetic bead depletions

PBMCs were suspended at 6 million cells/ml in 2% FBS/PBS (Bio-Whittaker). Magnetic beads (M-450 CD8 and M-450 CD4 DynaBeads; Dynal Biotech, Brown Deer, WI) were first washed in 2%FBS/PBS and used at an approximate 5X excess over the expected number of target cells. PBMCs and beads were rocked at 4°C for 20 minutes prior to magnetic separation (MPC-L, Dynal Biotech). The unbound PBMC fraction was recovered and subjected to a second magnetic separation to ensure removal of all beads. The cells were then transferred to a new tube, sedimented and suspended in RPMI-10 media. The cells were suspended at a concentration corresponding to 5 million PBMCs/ml based on the original input of PBMCs. The magnetic bead bound PBMC fraction was washed twice in 2%FBS/PBS and then suspended in a volume corresponding to 5 million input PBMCs/ml in RPMI-10 media.

##### Freezing

Cells were suspended at 10 million cells per ml in cold freezing medium (10% DMSO/90% FBS) and dispensed in 1 ml aliquots into 1.5 ml cryovials. The aliquots were placed into Nalgene Cryo 1°C Freezing container (Fisher Scientific, Pittsburgh, PA) in a -70°C freezer for 24 hrs before transfer to storage in liquid nitrogen.

### ELISPOT assay

The ELISPOT assay was carried out essentially as described[38]. Briefly, ELISPOT plates (Multiscreen-IP Filter plate, Millipore, Bedford, MA) were coated using antibody 1-D1K-γ, (MabTech AB, Sweden), and antibody 7-B6-1 (MabTech AB) as a secondary antibody. PBMCs (500,000/well) were added in RPMI-10 media. Peptides were added at concentrations of 0.33 ug/ml for CD4 cell assays and 3.3 ug/ml for CD8 cell assays. Concentrations represent the concentration of each individual peptide within the pool. Following a 24-hour incubation at 37°C in a CO_2 _incubator, ELISPOTS were developed and counted. All assays to screen T-cell responses were done with freshly isolated PBMCs. Assays where frozen samples were used are indicated in Figure legends.

### Tracking of CD8 cell depletion

PBMCs were recovered from whole blood and subjected to magnetic bead depletion of CD8 cells. Depletion was followed by fluorescence-activated cell sorter (FACS) analysis after staining cells with phycoerythrin-conjugated anti-human CD3 antibody (BD PharMingen, San Diego, CA) and fluorescein isothiocyanate (FITC)-conjugated anti-human CD8 antibody (BD PharMingen).

## Competing interests

The author(s) declare that they have no competing interests.

## References

[B1] Tao G, Kassler WJ, Rein DB (2000). Medical care expenditures for genital herpes in the United States. Sex Transm Dis.

[B2] Liesegang TJ (2001). Herpes simplex virus epidemiology and ocular importance. Cornea.

[B3] Tran T, Druce JD, Catton MC, Kelly H, Birch CJ (2004). Changing epidemiology of genital herpes simplex virus infection in Melbourne, Australia, between 1980 and 2003. Sex Transm Infect.

[B4] Roberts CM, Pfister JR, Spear SJ (2003). Increasing proportion of herpes simplex virus type 1 as a cause of genital herpes infection in college students. Sex Transm Dis.

[B5] Miller CS, Danaher RJ, Jacob RJ (1998). Molecular aspects of herpes simplex virus I latency, reactivation, and recurrence. Crit Rev Oral Biol Med.

[B6] Stanberry LR, Cunningham AL, Mindel A, Scott LL, Spruance SL, Aoki FY, Lacey CJ (2000). Prospects for control of herpes simplex virus disease through immunization. Clin Infect Dis.

[B7] Koelle DM, Corey L (2003). Recent progress in herpes simplex virus immunobiology and vaccine research. Clin Microbiol Rev.

[B8] Milligan GN, Dudley-McClain KL, Young CG, Chu CF (2004). T-cell-mediated mechanisms involved in resolution of genital herpes simplex virus type 2 (HSV-2) infection of mice. J Reprod Immunol.

[B9] Deshpande SP, Kumaraguru U, Rouse BT (2000). Why do we lack an effective vaccine against herpes simplex virus infections?. Microbes Infect.

[B10] Posavad CM, Koelle DM, Shaughnessy MF, Corey L (1997). Severe genital herpes infections in HIV-infected individuals with impaired herpes simplex virus-specific CD8+ cytotoxic T lymphocyte responses. Proc Natl Acad Sci U S A.

[B11] Khanna KM, Lepisto AJ, Decman V, Hendricks RL (2004). Immune control of herpes simplex virus during latency. Curr Opin Immunol.

[B12] Koelle DM, Chen HB, Gavin MA, Wald A, Kwok WW, Corey L (2001). CD8 CTL from genital herpes simplex lesions: recognition of viral tegument and immediate early proteins and lysis of infected cutaneous cells. J Immunol.

[B13] Koelle DM, Corey L, Burke RL, Eisenberg RJ, Cohen GH, Pichyangkura R, Triezenberg SJ (1994). Antigenic specificities of human CD4+ T-cell clones recovered from recurrent genital herpes simplex virus type 2 lesions. J Virol.

[B14] Mikloska Z, Ruckholdt M, Ghadiminejad I, Dunckley H, Denis M, Cunningham AL (2000). Monophosphoryl lipid A and QS21 increase CD8 T lymphocyte cytotoxicity to herpes simplex virus-2 infected cell proteins 4 and 27 through IFN-gamma and IL-12 production. J Immunol.

[B15] Tigges MA, Leng S, Johnson DC, Burke RL (1996). Human herpes simplex virus (HSV)-specific CD8+ CTL clones recognize HSV-2-infected fibroblasts after treatment with IFN-gamma or when virion host shutoff functions are disabled. J Immunol.

[B16] Keilholz U, Weber J, Finke JH, Gabrilovich DI, Kast WM, Disis ML, Kirkwood JM, Scheibenbogen C, Schlom J, Maino VC, Lyerly HK, Lee PP, Storkus W, Marincola F, Worobec A, Atkins MB (2002). Immunologic monitoring of cancer vaccine therapy: results of a workshop sponsored by the Society for Biological Therapy. J Immunother.

[B17] Tobery TW, Wang S, Wang XM, Neeper MP, Jansen KU, McClements WL, Caulfield MJ (2001). A simple and efficient method for the monitoring of antigen-specific T cell responses using peptide pool arrays in a modified ELISpot assay. J Immunol Methods.

[B18] Milligan GN, Bernstein DI (1997). Interferon-gamma enhances resolution of herpes simplex virus type 2 infection of the murine genital tract. Virology.

[B19] Liu T, Khanna KM, Carriere BN, Hendricks RL (2001). Gamma interferon can prevent herpes simplex virus type 1 reactivation from latency in sensory neurons. J Virol.

[B20] Khanna KM, Bonneau RH, Kinchington PR, Hendricks RL (2003). Herpes simplex virus-specific memory CD8+ T cells are selectively activated and retained in latently infected sensory ganglia. Immunity.

[B21] Liu T, Khanna KM, Chen X, Fink DJ, Hendricks RL (2000). CD8(+) T cells can block herpes simplex virus type 1 (HSV-1) reactivation from latency in sensory neurons. J Exp Med.

[B22] Koelle DM, Frank JM, Johnson ML, Kwok WW (1998). Recognition of herpes simplex virus type 2 tegument proteins by CD4 T cells infiltrating human genital herpes lesions. J Virol.

[B23] Byars NE, Fraser-Smith EB, Pecyk RA, Welch M, Nakano G, Burke RL, Hayward AR, Allison AC (1994). Vaccinating guinea pigs with recombinant glycoprotein D of herpes simplex virus in an efficacious adjuvant formulation elicits protection against vaginal infection. Vaccine.

[B24] Eisenberg RJ, Cerini CP, Heilman CJ, Joseph AD, Dietzschold B, Golub E, Long D, Ponce de Leon M, Cohen GH (1985). Synthetic glycoprotein D-related peptides protect mice against herpes simplex virus challenge. J Virol.

[B25] Rammensee HG, Falk K, Rotzschke O (1993). Peptides naturally presented by MHC class I molecules. Annu Rev Immunol.

[B26] Currier JR, Kuta EG, Turk E, Earhart LB, Loomis-Price L, Janetzki S, Ferrari G, Birx DL, Cox JH (2002). A panel of MHC class I restricted viral peptides for use as a quality control for vaccine trial ELISPOT assays. J Immunol Methods.

[B27] Looker KJ, Garnett GP (2005). A systematic review of the epidemiology and interaction of herpes simplex virus types 1 and 2. Sex Transm Infect.

[B28] Corey L, Langenberg AG, Ashley R, Sekulovich RE, Izu AE, Douglas JMJ, Handsfield HH, Warren T, Marr L, Tyring S, DiCarlo R, Adimora AA, Leone P, Dekker CL, Burke RL, Leong WP, Straus SE (1999). Recombinant glycoprotein vaccine for the prevention of genital HSV-2 infection: two randomized controlled trials. Chiron HSV Vaccine Study Group. Jama.

[B29] Bernstein DI, Aoki FY, Tyring SK, Stanberry LR, St-Pierre C, Shafran SD, Leroux-Roels G, Van Herck K, Bollaerts A, Dubin G (2005). Safety and immunogenicity of glycoprotein D-adjuvant genital herpes vaccine. Clin Infect Dis.

[B30] Koelle DM, Liu Z, McClurkan CL, Cevallos RC, Vieira J, Hosken NA, Meseda CA, Snow DC, Wald A, Corey L (2003). Immunodominance among herpes simplex virus-specific CD8 T cells expressing a tissue-specific homing receptor. Proc Natl Acad Sci U S A.

[B31] Simmons A, Tscharke DC (1992). Anti-CD8 impairs clearance of herpes simplex virus from the nervous system: implications for the fate of virally infected neurons. J Exp Med.

[B32] Koelle DM, Liu Z, McClurkan CM, Topp MS, Riddell SR, Pamer EG, Johnson AS, Wald A, Corey L (2002). Expression of cutaneous lymphocyte-associated antigen by CD8(+) T cells specific for a skin-tropic virus. J Clin Invest.

[B33] Theil D, Derfuss T, Paripovic I, Herberger S, Meinl E, Schueler O, Strupp M, Arbusow V, Brandt T (2003). Latent herpesvirus infection in human trigeminal ganglia causes chronic immune response. Am J Pathol.

[B34] Feldman LT, Ellison AR, Voytek CC, Yang L, Krause P, Margolis TP (2002). Spontaneous molecular reactivation of herpes simplex virus type 1 latency in mice. Proc Natl Acad Sci U S A.

[B35] Yasukawa M, Yakushijin Y, Furukawa M, Fujita S (1993). Specificity analysis of human CD4+ T-cell clones directed against human herpesvirus 6 (HHV-6), HHV-7, and human cytomegalovirus. J Virol.

[B36] Mikloska Z, Cunningham AL (1998). Herpes simplex virus type 1 glycoproteins gB, gC and gD are major targets for CD4 T-lymphocyte cytotoxicity in HLA-DR expressing human epidermal keratinocytes. J Gen Virol.

[B37] Koelle DM, Posavad CM, Barnum GR, Johnson ML, Frank JM, Corey L (1998). Clearance of HSV-2 from recurrent genital lesions correlates with infiltration of HSV-specific cytotoxic T lymphocytes. J Clin Invest.

[B38] Arrington J, Braun RP, Dong L, Fuller DH, Macklin MD, Umlauf SW, Wagner SJ, Wu MS, Payne LG, Haynes JR (2002). Plasmid vectors encoding cholera toxin or the heat-labile enterotoxin from Escherichia coli are strong adjuvants for DNA vaccines. J Virol.

